# Nickel induces inflammatory activation via NF-κB, MAPKs, IRF3 and NLRP3 inflammasome signaling pathways in macrophages

**DOI:** 10.18632/aging.102570

**Published:** 2019-12-10

**Authors:** Hongrui Guo, Huan Liu, Zhijie Jian, Hengmin Cui, Jing Fang, Zhicai Zuo, Junliang Deng, Yinglun Li, Xun Wang, Ling Zhao, Yi Geng, Ping Ouyang, Weiming Lai, Zhengli Chen, Chao Huang

**Affiliations:** 1 College of Veterinary Medicine, Sichuan Agricultural University, Wenjiang, Chengdu 611130, China; 2 Key Laboratory of Animal Diseases and Environmental Hazards of Sichuan Province, Sichuan Agriculture University, Wenjiang, Chengdu 611130, China; 3 Key Laboratory of Agricultural Information Engineering of Sichuan Province, Sichuan Agriculture University, Yaan, Sichuan 625014, China

**Keywords:** NiCl2, NF-κB, inflammasome, apoptosis, BMDMs

## Abstract

Nickel (Ni), an environmental hazard, widely causes allergic contact hypersensitivity worldwide. Despite that Ni-stimulated pro-inflammatory response is vital in allergy, the underlying molecular mechanisms remain largely unclear. Here, we demonstrated that NiCl_2_ activated nuclear factor kappa B (NF-κB), mitogen-activated protein kinases (MAPKs) and interferon regulatory factor 3 (IRF3) signaling pathways in primary bone marrow-derived macrophages (BMDMs), leading to the altered transcription levels of interleukin-1β (IL-1β), -6, -8, -18, tumor necrosis factor-α (TNF-α) and interferon β (INF-β). We also found that nickel chloride (NiCl_2_) activated Nod-like receptor 3 (NLRP3) inflammasome pathway, resulting in the proteolytic cleavage and release of IL-1β. NiCl_2_ induced the accumulation of mitochondrial reactive oxygen species (mtROS) and the release of mitochondrial DNA (mtDNA), thus activating NLRP3 inflammasome pathway. Additionally, NiCl_2_-induced apoptosis was dependent on the generation of mtROS, and caspase-1 activation might also partly contribute to the apoptotic process. Altogether, abovementioned results indicate that NiCl_2_ induces inflammatory activation in BMDMs via NF-κB, MAPKs, IRF3 signaling pathways as well as NLRP3 inflammasome pathway, which provides a mechanism to improve the efficiency of treatment against Ni-induced allergic reactions.

## INTRODUCTION

Nickel (Ni) has been recognized as a ubiquitous environmental contaminant, which is commonly used in electronic processing and medical industries, such as electroplating and the manufacturing processes of battery, electronic device and stainless steel [1]. Several epidemiological reports have indicated that occupational Ni exposure is associated with a higher prevalence of human nasal and lung cancers [2, 3]. Many other studies have suggested that Ni or Ni compounds can induce carcinogenicity, cytotoxicity, genotoxicity, immunotoxicity and mutagenicity both *in vivo* and *in vitro* [4, 5]. The broad application of Ni has resulted in its elevated levels in biogeochemical cycles, and increased its environmental exposure in humans [6].

At present, Ni-containing alloys are commonly used as biomaterials for cardiovascular, dental and orthopedic applications [7, 8]. It has been reported that Ni^2+^ is released from Ni-alloy during corrosion process [9, 10]. Ni^2+^ is released not only in medical devices such as dental restorations, surgical instruments, orthopedic implants and vascular stents, but also from coins, jewelry, mobile phones, piercing materials and synthetic nanoparticles [11]. Ni^2+^ is among the most frequent causes of allergic contact dermatitis in humans [12], which can affect the local and systemic immunity by suppressing the immune system or activating different inflammatory mediators such as intracellular adhesion molecule 1 and pro-inflammatory cytokines [13].

Inflammation represents cellular responses to infection, stress or injury [14]. Ni^2+^ can directly activate pro-inflammatory intracellular signal transduction cascades that stimulate mitogen-activated protein kinase (MAPK) p38 and nuclear factor-κB (NF-κB) [15]. It has been also shown that Ni and Ni compounds can induce the up-regulation of interleukin-1β (IL-1β), -6, -8, -18, tumor necrosis factor-α (TNF-α) and cyclooxygenase-2 (COX-2) [16, 17]. The maturation and release of IL-1β and IL-18 in macrophages are regulated by an inflammatory signaling platform, namely, “inflammasome” [18]. Inflammasome triggers the activation of caspase-1 in responses to cellular stresses and pathogenic infections [19]. The most studied inflammasome is Nod-like receptor 3 (NLRP3) inflammasome, which can be activated by numerous stimuli including infection and metabolic disorders [20]. NLRP3 inflammasome contains three subunits: NLRP3; caspase-1, the effector subunit; and apoptosis-associated speck-like protein containing a CARD (ASC) [20]. Although it is not completely clear, mitochondrial ROS generation and mtDNA release are the plausible stimulators for the production of NLRP3 inflammasome [18, 21]. Recruitment of caspase-1 into the inflammasome complexes can result in its full activation, auto-processing and substrate cleavage. Additionally, few studies have shown that Ni^2+^ can activate inflammasome pathway [22, 23].

In recent years, substantial attention has been paid to Ni uptake by macrophages [24–26]. Besides, direct T cell interaction or soluble mediator release can modulate the responses of macrophages to metal-containing alloys [27]. Here, we investigated the mechanism of nickel chloride (NiCl_2_)-induced inflammatory response, such as NF-κB, MAPKs and interferon regulatory factor 3 (IRF3) signaling pathways as well as NLRP3 inflammasome pathway in bone marrow-derived macrophages (BMDMs). Our findings would reveal a novel molecular mechanism underlying NI-induced inflammatory responses, which can improve future therapies against Ni-induced allergic reactions.

## RESULTS

### NiCl_2_ induces cytotoxicity in BMDMs

**Figure 1:**
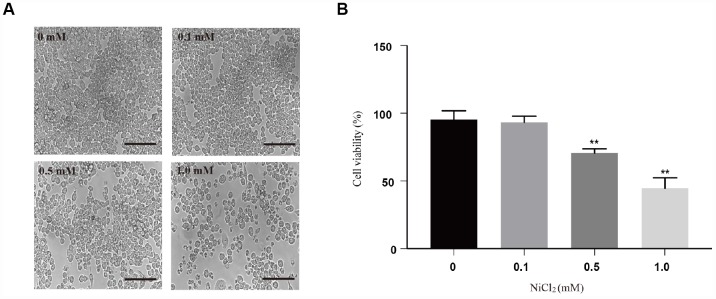
**Cytotoxicity of NiCl_2_ in BMDMs.** (**A**) BMDMs are treated with NiCl_2_ (0, 0.1, 0.5 and 1.0 mM) for 24h, and changes of cell numbers were observed by microscopy. Scale bar 50 μm. (**B**) Cell viability is analyzed by MTT assay. Data are presented with the means ± standard deviation (n=5). *p < 0.05 and **p < 0.01, compared with the control group.

Our previous work has shown that NiCl_2_ can inhibit the immune response in chicken. To evaluate the cytotoxicity of NiCl_2_, BMDMs were exposed to various doses of NiCl_2_ (0, 0.1, 0.5 and 1.0 mM) for 24 h. It was found that NiCl_2_ suppressed BMDMs viability in a dose-dependent manner (Figure 1A and 1B). Notably, the viabilities of BMDMs were significantly (p < 0.01) decreased in 0.5 and 1.0 mM NiCl_2_ exposure groups compared to control group.

### NiCl_2_ activates NF-κB, MAPKs and IRF3 pathways in BMDMs

**Figure 2:**
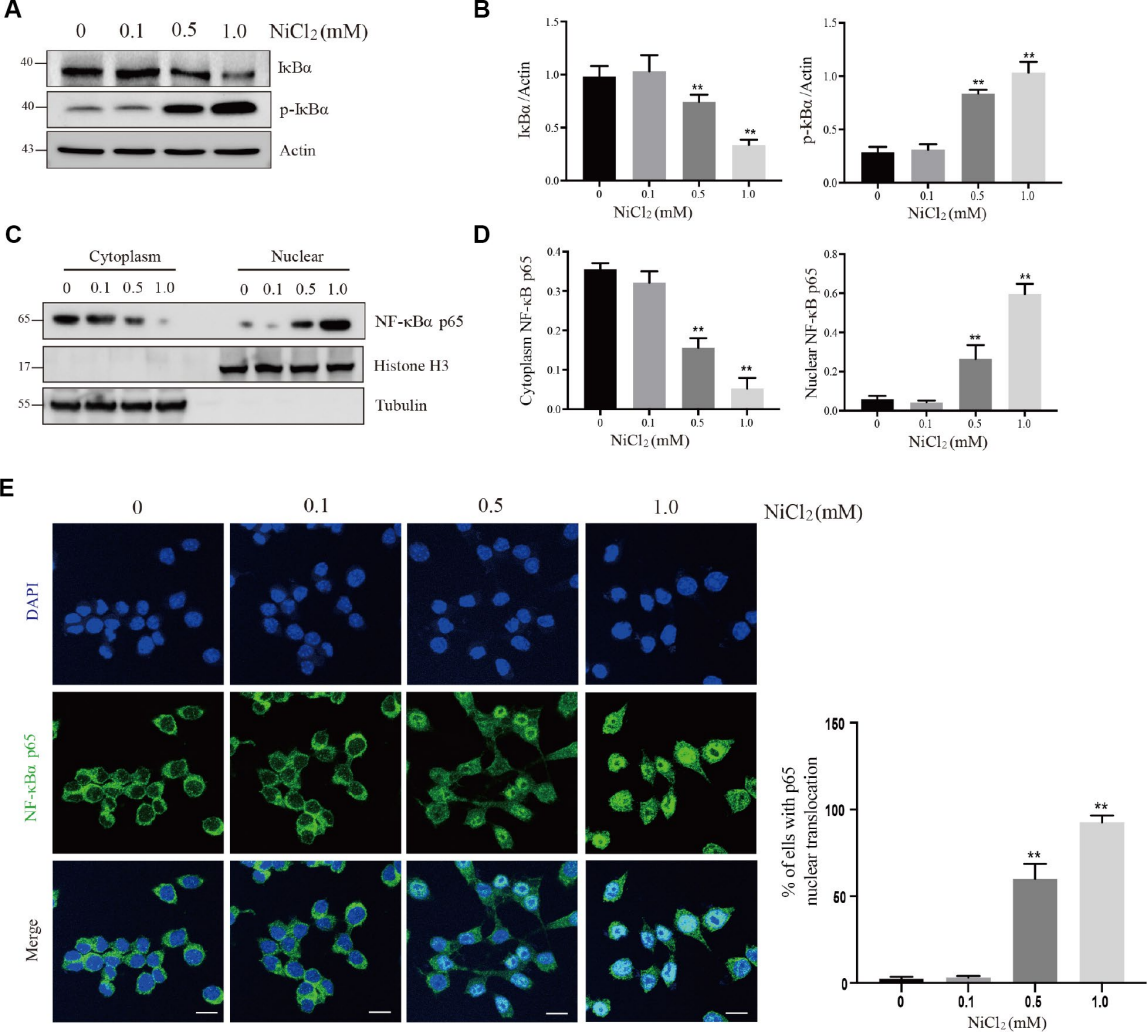
**NiCl_2_ activates NF-κB pathway in BMDMs.** (**A** and **B**) BMDMs are treated with NiCl_2_ (0, 0.1, 0.5 and 1.0 mM) for 24h, and immunoblotted for the whole cell lysis IκBα and p-IκBα protein expression. (**C** and **D**) cells are treated with NiCl_2_ (0, 0.1, 0.5 and 1.0 mM) for 24h, and immunoblotted for the cytoplasm and nuclear cell lysis NF-κB p65 protein expression. (**E**) The translocation of NF-κB p65 protein determined by NF-κB p65 staining of NiCl_2_-primed BMDMs. Scale bar 50 μm. Data are presented with the means ± standard deviation (n=5). *p < 0.05 and **p < 0.01, compared with the control group.

To investigate the inflammatory potential of NiCl_2_, NF-κB pathway was tested. The activation of NF-κB transcription factor may trigger an inflammatory response [15]. NF-κB proteins are bound and inhibited by IκBα proteins. NiCl_2_ treatment increased the phosphorylation levels of IκBα protein expression, and decreased the total IκBα protein expression levels (Figure 2A and 2B). Phosphorylation of IκBα caused its ubiquitination and proteasomal degradation, freeing NF-κB complexes. In Figure 2C and 2E, the results showed that NF-κB was translocated to the nucleus, and thus inducing target gene expression.

**Figure 3:**
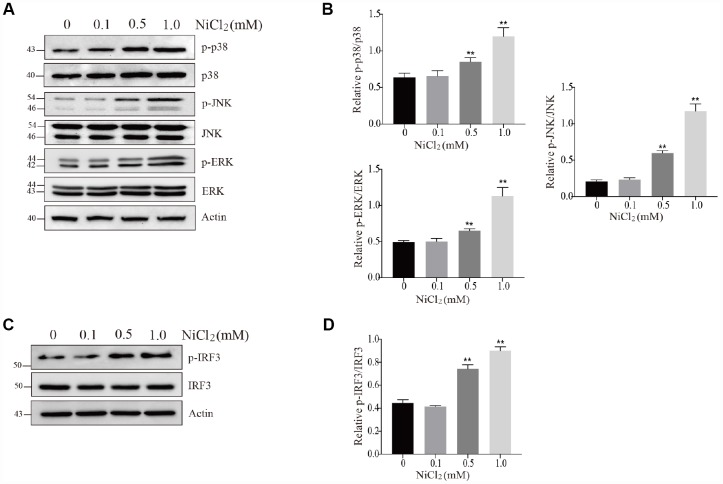
**NiCl_2_ activates MAPKs and IRF3 pathway in BMDMs.** (**A** and **B**) BMDMs are treated with NiCl_2_ (0, 0.1, 0.5 and 1.0 mM) for 24h, and immunoblotted for the whole cell lysis p-p38, p38, p-JNK, JNK, p-ERK and ERK protein expression. (**C** and **D**) BMDMs are treated with NiCl_2_ (0, 0.1, 0.5 and 1.0 mM) for 24h, and immunoblotted for the p-IRF3 and IRF3 protein expression. Data are presented with the means ± standard deviation (n=5). *p < 0.05 and **p < 0.01, compared with the control group.

ERK1/2, Jun amino-terminal kinases (JNK) and p38 play crucial roles in cell growth and differentiation as well as inflammatory response and cellular stress [28]. In Figure 3A and 3B, our results showed that the protein expression levels of p-ERK, p-JNK and p-p38 were remarkably (p < 0.01) increased in 0.5 and 1.0 mM NiCl_2_ exposure groups.

Interferon regulatory factor 3 (IRF3) can suppress cell proliferation and regulate the expression levels of genes related to innate immunity [29]. As shown in Figure 3C and 3D, NiCl_2_ exposure markedly (p < 0.01) increased the protein expression levels of p-IRF3.

### NiCl_2_ induces pro-inflammatory cytokine production in BMDMs

**Figure 4:**
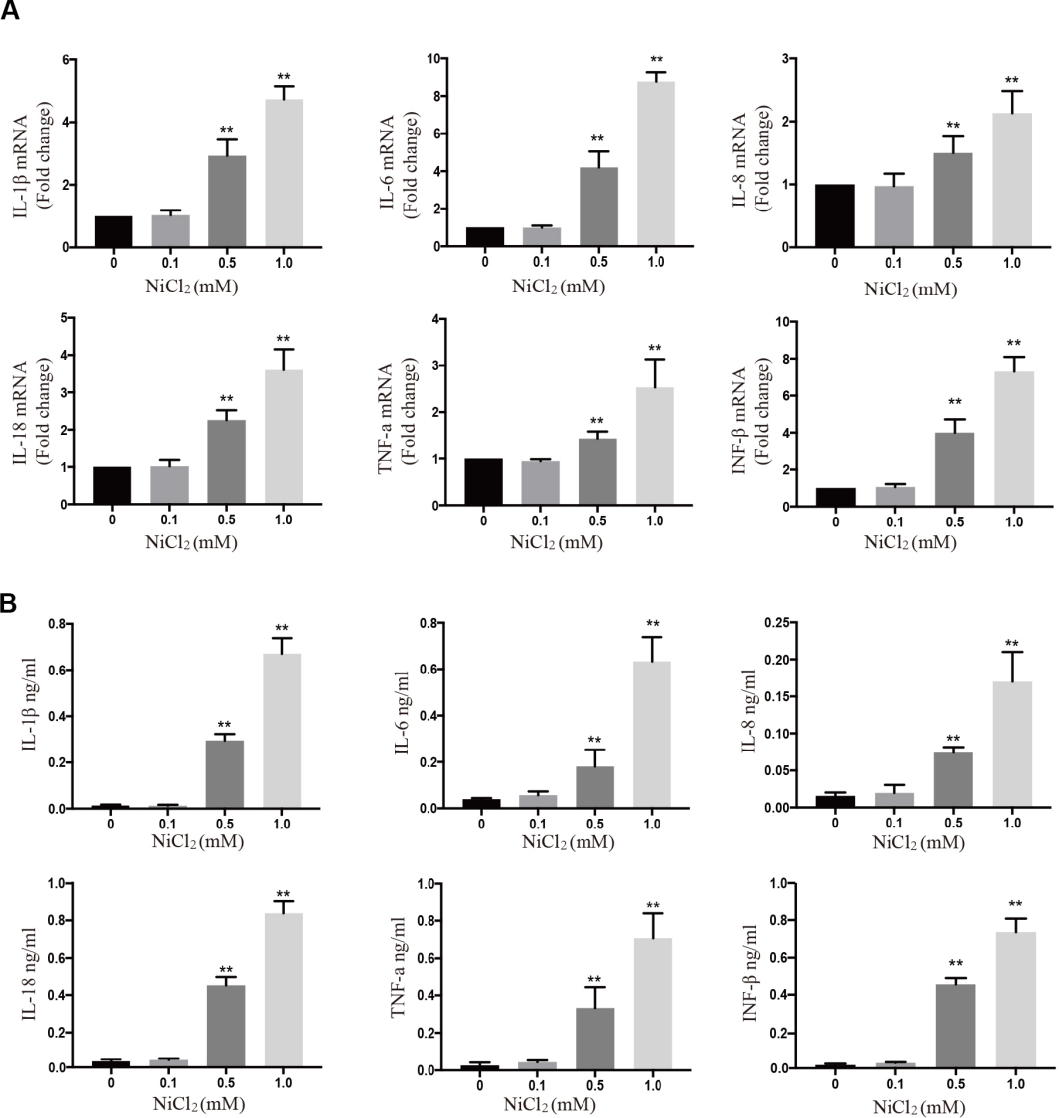
**NiCl_2_ induces pro-inflammatory cytokine production in BMDMs.** (**A**) The mRNA expression levels of inflammatory cytokines after NiCl_2_ (0, 0.1, 0.5 and 1.0 mM) treatment for 24h. (**B**) Inflammatory cytokine expression is quantified in BMDMs cultural medium by ELISA after NiCl_2_ (0, 0.1, 0.5 and 1.0 mM) treatment for 24h. Data are presented with the means ± standard deviation (n=5). *p < 0.05 and **p < 0.01, compared with the control group.

The above results showed that NiCl_2_ activated NF-κB, MAPKs and IRF3 pathways, by upregulating the expression levels of pro-inflammatory cytokines. In Figure 4A, the mRNA expression levels of IL-1β, -6, -8, -18, TNF-α and IFN-β were remarkably (p < 0.01) increased in 0.5 and 1.0 mM NiCl_2_ exposure groups compared to those in control group. The ELISA data revealed that NiCl_2_ treatment markedly (p < 0.01) increased the production of IL-1β, -6, -8, -18, TNF-α and IFN-β (Figure 4B).

### NiCl_2_ activates NLRP3 inflammasome pathway in BMDMs

**Figure 5:**
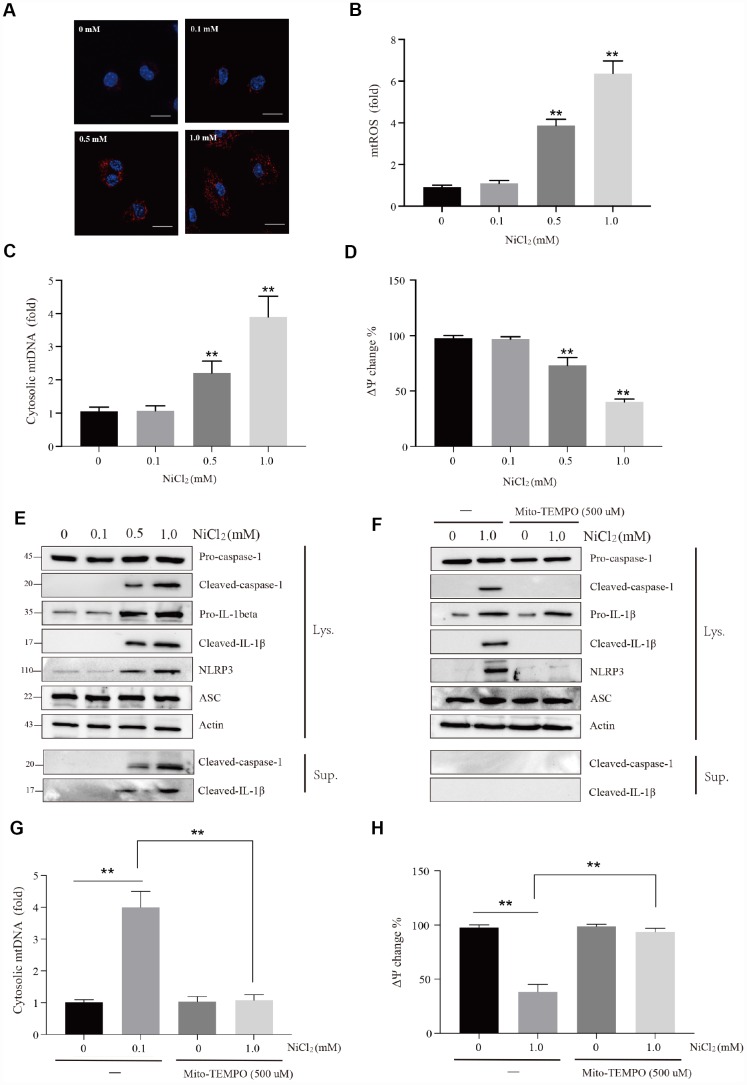
**NiCl_2_ activates NLRP3 inflammasome pathway in BMDMs.** (**A** and **B**) Relative mtROS amounts determined by MitoSOX-red staining of NiCl_2_-primed BMDMs. Scale bar 50 μm. (**C**) Relative cytosolic mtDNA expression in NiCl_2_-primed BMDMs. (**D**) NiCl_2_-induced changes in mitochondrial membrane potential (Ψm) in BMDMs measured by TMRM fluorescence. (**E**) Immunoblot analysis of pro-caspase-1, cleaved-caspase-1, pro-IL-1β, cleaved- IL-1β, NLRP3 and ASC in lysates of NiCl_2_-treated BMDMs, and cleaved-caspase-1and cleaved- IL-1β in the supernatant. (**F**) Immunoblot analysis of pro-caspase-1, cleaved-caspase-1, pro-IL-1β, cleaved- IL-1β, NLRP3 and ASC in lysates of Mito-TEMPO (500 μM)-pre-treated 1h before 24h of NiCl_2_ stimulation. (**G**) Relative cytosolic mtDNA expression in NiCl_2_-treated (24h) BMDMs in the presence/absence of Mito-TEMPO (500 μM, 1h) pre-treatment. (**H**) Changes of mitochondrial membrane potential (Ψm) in NiCl_2_-treated (24h) BMDMs in the presence/absence of Mito-TEMPO (500 μM, 1h) pre-treatment. Data are presented with the means ± standard deviation (n=5). *p < 0.05 and **p < 0.01, compared with the control group.

NiCl_2_ activated caspase-1-mediated inflammatory cytokine production in BMDMs. Inflammasomes are multi-protein signaling complexes that promote inflammatory caspases activation and IL-1β maturation. We tested whether NLRP3-ASC-caspase-1 inflammasome is activated after NiCl_2_ treatment. It was found that NiCl_2_ induced the cleavage of IL-1β and caspase-1 (Figure 5E).

The mechanism underlying NiCl_2_-induced caspase-1 activation was explored in this study. The results showed that NiCl_2_ induced mitochondria damage such as increase of mitochondrial ROS (mtROS) production (Figure 5A and 5B), elevation of mitochondrial DNA (mtDNA) release (Figure 5C) and decrease of mitochondrial membrane potential (Figure 5D). We further assessed whether mitochondria-specific ROS production is associated with NiCl_2_-induced activation of caspase-1. Mito-TEMPO, a mitochondria-specific ROS scavenger, suppressed the formation of cleaved-IL-1β and cleaved-caspase-1 in BMDMs in response to NiCl_2_ (Figure 5F). Additionally, Mito-TEMPO attenuated the elevation of mtDNA release and loss of mitochondrial membrane potential (Figure 5G and 5H). These data demonstrate that NiCl_2_-induced caspase-1 activation largely relies on the mitochondrial generation of ROS.

### NiCl_2_ induces apoptosis in BMDMs

**Figure 6:**
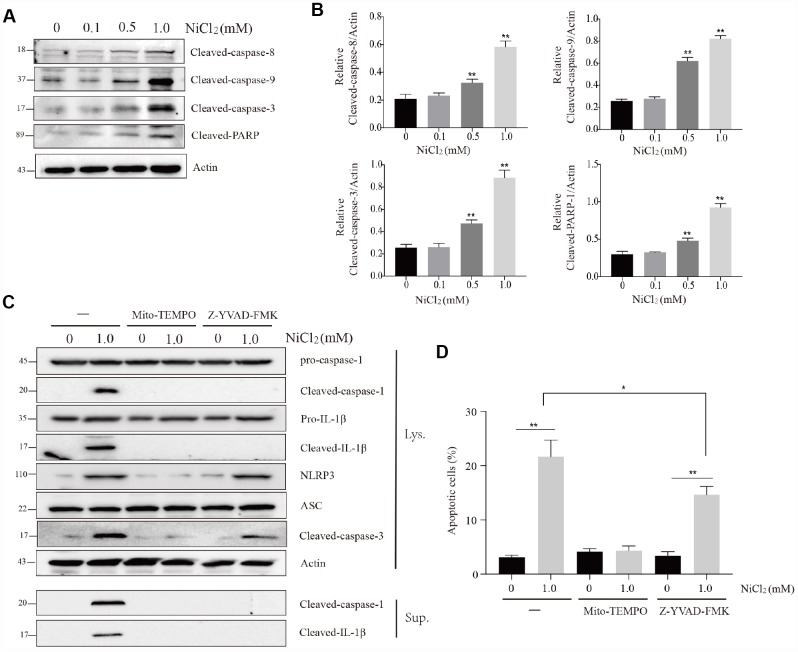
**NiCl_2_ induces apoptosis in BMDMs.** (**A** and **B**) Immunoblot analysis of cleaved-caspase-8, cleaved-caspase-9, cleaved-caspase-3 and cleaved-PARP in lysates of the NiCl_2_-treated BMDMs. (**C**) Immunoblot analysis of pro-caspase-1, cleaved-caspase-1, pro-IL-1β, cleaved- IL-1β, NLRP3, ASC and cleaved-caspase-3 in the NiCl_2_-treated (24h) BMDMs in the presence/absence of Mito-TEMPO (500 μM, 1h) or Z-YVAD-FMK (100 μM, 1h) pre-treatment, and cleaved-caspase-1and cleaved- IL-1β in the supernatant. (**D**) Flow cytometry analysis apoptosis in the NiCl_2_-treated (24h) BMDMs in the presence/absence of Mito-TEMPO (500 μM, 1h) or Z-YVAD-FMK (100 μM, 1h) pre-treatment. Data are presented with the means ± standard deviation (n=5). *p < 0.05 and **p < 0.01, compared with the control group.

In Figure 6A and 6B, we observed that the protein expression levels of cleaved-caspase-3, -8, -9 and cleaved-poly (ADP-ribose) polymerase (PARP) were significantly (p < 0.01) increased in 0.5 and 1.0 mM NiCl_2_ exposure groups (Figure 6A and 6B).

MtROS generation diminishes mitochondrial membrane potential, and subsequently induces mitochondria-mediated apoptosis. Our above-mentioned results showed that NiCl_2_ treatment resulted in excessive mitochondrial ROS production and decreased mitochondrial membrane potential. Next, we examined whether mitochondrial ROS and caspase-1 activation are involved in NiCl_2_-induced apoptosis.

Cotreatment with NiCl_2_ and Mito-TEMPO suppressed the cleavage of caspase-1, -3 and IL-1β (Figure 6C). Cotreatment with NiCl_2_ and Z-YVAD-FMK, a potent inhibitor of caspase-1, suppressed the cleavage of IL-1β and caspase-1 (Figure 6C). However, the caspase-1 inhibitor only partly inhibited the cleaved-caspase-3 protein expression (Figure 6C). The percentage of apoptosis was significantly (p < 0.01) higher in NiCl_2_ treatment groups and NiCl_2_+Z-YVAD-FMK treatment group than that in control group (Figure 6D).

Taken altogether, these findings indicate that NiCl_2_-induced apoptosis is dependent on mitochondrial ROS production, and caspase-1 activation may also partly contribute to the apoptotic process.

## DISCUSSION

In the present study, our findings indicated that NiCl_2_ was extremely toxic to macrophages *in* *vitro.* In addition, our results demonstrated that the excessive inflammatory responses and apoptosis were both associated with NiCl_2_ toxicity in BMDMs.

In our previous studies, we have found that NiCl_2_ induces inflammatory destruction in the kidney and liver of broiler chickens, by activating NF-κB pathway activation and upregulating the mRNA levels of IL-1β, -6, -8 and TNF-α [30, 31]. Here, we showed that NiCl_2_ activated NF-κB pathway. Typically, NF-κB sequestration is initiated by IκB in the cytoplasm. IκB degradation may translocate NF-κB into the nucleus from the cytoplasm (Figure 2C), where it regulates the transcription of specific genes encoding inducible enzymes, pro-inflammatory cytokines and chemokines. After NiCl_2_ treatment, the mRNA and protein levels of IL-1β, -6, -8, -18, TNF-α and INF-β were remarkably increased (Figure 3). Further, we investigated whether MAPKs and IRF3 pathways are also activated in BMDMs following NiCl_2_ treatment. Indeed, NiCl_2_ treatment results in the phosphorylation of p38, JNK, ERK and IRF3, which translocates to the nucleus in order to activate its binding site-containing promoter regions, and then upregulates the transcription of pro-inflammatory cytokines. Besides, it has been demonstrated that nickel oxide nanoparticles (NiONPs) activate MAPKs (p38 and JNK) and NF-κB pathways in lung-derived A549 and BEAS-2B cell lines [16].

In macrophages, inflammasome controls the maturation and release of IL-1β [20, 32]. In this study, NiCl_2_ upregulated the mRNA and protein levels of IL-1β via NF-κB, MAPKs and IRF3 signaling pathways, and enhanced the maturation and production of IL-1β through NLRP3 inflammasome induction. Li and colleagues [22] have demonstrated that Ni induces the secretion of IL-1β via NLRP3-ASC-caspase-1 pathway. In addition, a previous study has shown that the induction of NLRP3 inflammasome largely depends on mitochondrial damage [18]. In this study, we observed increased mtROS generation and reduced mitochondrial membrane potential (Ψm). As a consequence of mtROS accumulation and mitochondrial membrane potential (Ψm), we found that NiCl_2_ treatment released mtDNA into the cytosolic compartment. Next, we tested whether mtROS generation is a relatively upstream step during NiCl_2_-induced NLRP3 inflammasome. Treatment with Mito-TEMPO (mitochondrial ROS scavenger) abolished the activation of NLRP3 inflammasome, such as the inhibition of cleaved-caspase-1 and IL-1β. Mito-TEMPO also suppressed mtDNA release and loss of mitochondrial membrane potential (Ψm). It has been demonstrated that mtROS generation is critical for the stimulation of NLRP3 inflammasome [33, 34]. Nonetheless, there is a report that mtDNA plays a pivotal role during inflammasome activation [35]. Collectively, our results indicate that NiCl_2_ induces NLRP3 inflammasome activation through mtROS production.

In this study, NiCl_2_ also induces the apoptosis of BMDMs, and macrophages are critical components of both innate and adaptive immunity, suggesting that NiCl_2_-induced BMDM apoptosis inhibits the immune function. In the three NiCl_2_ treatment groups, the transcription levels cleaved-caspase-3, -8, -9 and cleaved-PARP were upregulated. Furthermore, Mito-TEMPO could abolish the enhance levels of cleaved-caspase-3 and BMDM apoptosis. The above-mentioned results show that NiCl_2_ triggers mtROS generation and loss of mitochondrial membrane potential (Ψm). Taken together, NiCl_2_ aggravates BMDM apoptosis via mitochondrial ROS-associated pathway. These findings are consistent with those of Zuo et al. [36], in which nickel sulfate induces apoptosis in rat Leydig cells through activation of ROS-dependent mitochondria pathway. Likewise, the data of this study are compatible with our previous work that NiCl_2_ induces apoptosis in the kidney of broiler chickens via mitochondria-dependent apoptotic pathway [37]. Interestingly, Z-YVAD-FMK (caspase-1 inhibitor) can decrease at least part of the apoptotic process. Thus, NiCl_2_-induced NLRP3 inflammasome activation may contribute to cell apoptosis.

**Figure 7:**
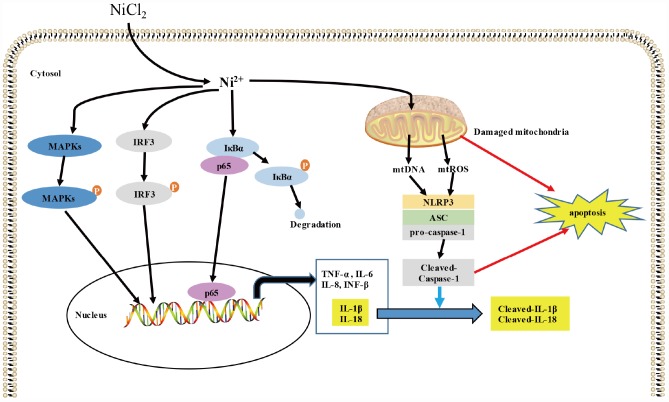
**Schematic diagram of the possible inflammatory response induced by NiCl_2_.** NiCl_2_ triggers the transcription of pro-inflammatory cytokines, including IL-1β, IL-6, IL-8, IL-18, TNF-α and INF-β through the NF-κB, MAPKs, IRF3 signaling pathways in the BMDM. And NiCl_2_ also can induce caspase-1 activation via NRLP3 inflammasome activation. Moreover, NiCl_2_ induces apoptosis through mitochondrial ROS-mediated pathway, and also, NLRP3 inflammasome activation contributes to the apoptosis.

In summary, this study reveals that NiCl_2_ triggers the transcription levels of pro-inflammatory cytokines, including IL-1β, -6, -8, -18, TNF-α and INF-β in BMDMs through NF-κB, MAPKs and IRF3 signaling pathways. Moreover, NiCl_2_ stimulates caspase-1 activation via NLRP3 inflammasome activation. Furthermore, NiCl_2_ induces apoptosis through mitochondrial ROS-mediated pathway, and the activation of NLRP3 inflammasome can contribute to apoptotic process (as shown in the Figure 7).

## MATERIALS AND METHODS

### Materials and reagents

NiCl_2_ (451193) was purchased from Sigma Aldrich Corporation. The mitochondria-targeted antioxidant Mito-TEMPO (CAS 1569257-94-8) was bought from Santa Cruz Biotechnology. Tetramethylrhodamine, methyl ester (TMRM) was obtained from AnaSpec Inc. (#CA94555). Z-YVAD-FMK (ALX-260-154-R100) was purchased from Enzo Life Sciences. MitoSOX (M36008) was obtained from Invitrogen.

The antibodies such as mouse anti-IκBα (#4814, 1:1000 for WB), rabbit anti-phospho (p)-IκBα (#2859, 1:1000 for WB), rabbit anti-NF-κB p65 (#8242, 1:1000 for WB, 1:200 for IF), mouse anti-β-Actin (#3700, 1:1000 for WB), rabbit anti-p38 (#8690, 1:1000 for WB), rabbit anti-p-p38 (#4511, 1:1000 for WB), rabbit anti-JNK (#9252, 1:1000 for WB), rabbit anti-p-JNK (#4668, 1:1000 for WB), rabbit anti-ERK (#4695, 1:1000 for WB), rabbit anti-p-ERK (#9101, 1:1000 for WB), rabbit anti-IRF3 (#11904, 1:1000 for WB), rabbit anti-p-IRF3 (#83611, 1:1000 for WB), rabbit anti-NLRP3 (#15101, 1:1000 for WB), rabbit anti-IL-1β (#12426S, 1:1000 for WB), rabbit anti-cleaved-caspase-3 (#9664, 1:1000 for WB), rabbit anti-cleaved-caspase-8 (#8592, 1:1000 for WB), rabbit anti-cleaved-caspase-9 (#9509, 1:1000 for WB), rabbit anti-PARP (#9548, 1:1000 for WB) were supplied by Cell Signaling Technology. Mouse anti-Histone H3 (sc-517576, 1:1000 for WB) and mouse anti-Tubulin (sc-73242, 1:1000 for WB) were obtained from Santa Cruz Biotechnology, while mouse anti-caspase-1 p20 (#AG-20B-0042-C100, 1:1000 for WB) and mouse anti-ASC (#AG-25B-0006-C100, 1:1000 for WB) were purchased from Adipogen.

IL-1β (MLB00C), IL-6 (M6000B), IL-18 (7625), TNF-α (MTA00B) and IFN-β (42400-1) ELISA kits were purchased from R&D system, while IL-8 (MBS261967) ELISA kit was obtained from MyBioSource.

### Cell isolation and culture

Preparation of primary BMDM was carried out as proposed by Nakahira et al. [35]. Briefly, BMDMs were collected from the femur and tibia of each mouse, and seeded on a sterile petri dish. The BMDMs were cultured for 7 days in DMEM supplemented with 10% heat-inactivated fetal bovine serum, penicillin, streptomycin and 25% conditioned medium from L929 mouse fibroblasts. Upon reaching confluency, the BMDMs were exposed to different concentrations (0, 0.1, 0.5 and 1.0 mM) of NiCl_2_ for 24 h. In the cotreatment with NiCl_2_ and Mito-TEMPO experiment, the Mito-TEMPO (500 μM) or Z-YVAD-FMK (100 μM) were treated 1h before 24h of NiCl_2_ treatment. In the cotreatment with NiCl_2_ and Z-YVAD-FMK experiment, the Z-YVAD-FMK (100 μM) were treated 1h before 24h of NiCl_2_ treatment.

### Cytotoxicity assessment

The viability of BMDMs was assessed using MTT assay. Briefly, BMDMs (1 × 10^6^ cells/ml) were suspended in complete culture media, and 150 μL of the cell suspension was cultured 48-well plates for 24 h. After treatment with NiCl_2_, 0.5 mg/ml MTT solution was added onto the BMDMs, followed by a 4 h incubation period. The obtained formazan crystals were dissolved in dimethyl sulfoxide, and the absorbance was recorded at 540 nm using a microplate reader (PerkinElmer). Data were the mean ± SD of 5 independent experiments conducted in triplicate.

### Western blotting

After NiCl_2_ treatment, cell lysis was performed with ice-cold RIPA buffer. After centrifuging at 15000 g for 15 min at 4 °C, the protein lysates were separated by 12% SDS-PAGE and then transferred onto PVDF membranes. Subsequently, the membranes were blocked with 5% non-fat milk in TBST, and then incubated with primary antibodies, followed by incubation with horseradish peroxidase (HRP)-conjugated secondary antibodies and enhanced chemiluminescent (ECL) detection (GE Healthcare, Piscataway, NJ, USA). The protein bands were visualized using Bio-Rad ChemiDoc XRS+ System (Bio-Rad Laboratories, Inc., Hercules, CA, USA). The statistical differences in protein expression levels were computed by an ImageJ2x software.

The supernatant protein is extracted by the method of trichloroacetic acid (TCA) precipitation of proteins according to the reference [38], followed by the detection of protein content using BCA protein assay. The expression levels of cleaved-caspase-1 and IL-1β are detected by western blotting.

### Determination of NF-κB p65 protein expression

After treatment with NiCl_2_ for 24 h, BMDMs were collected and total protein was isolated from cytoplasm and nucleus by using Cytoplasmic and Nuclear Protein Extraction Kit (SINP001 Viagene Biotech). The protein expression was determined by Western blotting.

BMDMs were seeded and allowed to be adhered on a coverslip for 24 h. After exposure to NiCl_2_ for 24 h, the BMDMs were fixed and permeabilized with 4% paraformaldehyde and 0.05% Triton X-100, respectively. After blocking with PBS containing 5% BSA, the BMDMs were stained with rabbit anti-rabbit NF-κB p65, followed by Alexa Fluor 488-conjugated anti-rabbit IgG secondary antibody (Jackson ImmunoResearch). The nuclei of BMDMs were stained with propidium iodide (PI; Thermo Fisher). All images were acquired with a confocal microscope. For image quantification, 100 cells randomly chosen from 10 high-power fields and pooled from five independent experiments, were evaluated for the distribution pattern of the indicated molecules. Then, we quantified the percentage of cells with NF-kB nuclear translocation.

### Determination of mitochondrial ROS

MitoSOX was used to measure mtROS production in BMDMs. After NiCl_2_ treatment, the BMDMs were rinsed in PBS and then stained with 5 μM of MitoSOX-red at 37 °C, 5% CO_2_ for 15 min. After MitoSOX staining, the BMDMs were washed with PBS, followed by fixing and staining with DAPI. All samples were visualized and recorded using Zeiss Axio Imager A2 fluorescence microscope. For image quantification, 100 cells randomly chosen from 10 high-power fields and pooled from five independent experiments, were evaluated for the distribution pattern of the indicated molecules. The red fluorescence of each cell is detected by image J.

### Measurements of mitochondrial membrane potential (Ψm)

Mitochondrial membrane potential (Ψm) was measured using TMRM as proposed by Zhong et al. [39]. Briefly, after NiCl_2_ exposure, the BMDMs were rinsed twice in PBS, incubated with 200 nM of TMRM for 30 min, and then rinsed twice in 50 nM of TMRM. The BMDMs were resuspended in PBS, and the fluorescence intensities recorded using FilterMax F5 multimode plate reader (Molecular Devices).

### Cellular fractionation and determination of cytosolic mtDNA

MtDNA in cytosol was performed as described previously [35]. Total DNA was extracted from the cytosolic fraction (200 μL) using DNeasy Blood and Tissue kit (Cat. 69504 Qiagen). Quantitative PCR was adopted for the measurement of mtDNA by using the established mitochondrial and nuclear gene primers and SYBR® Premix Ex Taq^TM^II (RR820A, Takara, China). The copy numbers of mtDNA were normalized to those of nuclear DNA, and the data were expressed as the ratio of mtDNA (cytochrome c oxidase I) to nuclear DNA (18S rRNA). The accession number, sequences, product size and annealing temperature of each primer are presented in Table 1.

**Table 1:** Primer sequences of genes selected for analysis mtDNA.

**Target gene**	**Accession number**	**Primer**	**Primer sequence (5′-3′)**	**Product size**	**Tm (°C)**
18S	EU120032	Forward	TAGAGGGACAAGTGGCGTTC	104bp	60
		Reverse	CGCTGAGCCAGTCAGTGT		
mouse cytochrome c oxidase I	XM_006980540	Forward	GCCCCAGATATAGCATTCCC	221bp	59
		Reverse	GTTCATCCTGTTCCTGCTCC		

### RNA isolation and quantitative Real-Time PCR (qRT-PCR)

Total RNA was isolated from the treated BMDM and was reverse transcribed. QRT-PCR reactions were carried out as proposed by Guo et al. [30]. QRT-PCR data were analyzed by the 2^−ΔΔCT^ method [40]. The accession number, sequences, product size and annealing temperature of each primer are listed in Table 2.

**Table 2:** Primer sequences of genes selected for analysis pro-inflammatory cytokines.

**Target gene**	**Accession number**	**Primer**	**Primer sequence (5′-3′)**	**Product size**	**Tm (°C)**
IL-1β	NM_008361	Forward	AATGCCACCTTTTGACAGTGAT	132bp	60
		Reverse	TGCTGCGAGATTTGAAGCTG		
IL-6	NM_001314054	Forward	AGGATACCACTCCCAACAGACC	140bp	60
		Reverse	AAGTGCATCATCGTTGTTCATACA		
IL-8	NM_ 011339	Forward	TTTCCACCGGCAATGAAG	118bp	59
		Reverse	TAGAGGTCTCCCGAATTGGA		
IL-18	NM_008360	Forward	GGCTGCCATGTCAGAAGACT	239bp	60
		Reverse	GTCTGGTCTGGGGTTCACTG		
TNF-a	NM_013693	Forward	CACGTCGTAGCAAACCACC	88bp	59
		Reverse	TGAGATCCATGCCGTTGGC		
INF-β	NM_010510	Forward	GGCTTCCATCATGAACAACAGGT	167bp	61
		Reverse	AGGTGAGGTTGATCTTTCCATTCAG		
β-actin	NM_007393	Forward	GCTGTGCTATGTTGCTCTAG	117bp	59
		Reverse	CGCTCGTTGCCAATAGTG		

### Enzyme-linked immunosorbent assay

After NiCl_2_ treatment, ELISA kit was used to examine the concentration of cytokines in cell culture supernatants as per the manufacturer's protocols.

### Apoptosis analysis by flow cytometry

After NiCl_2_ treatment, the BMDMs were rinsed twice with ice-cold phosphate buffer saline (PBS, pH 7.2-7.4), and then suspended in PBS to the desired concentration of 1×10^6^ cells/ml. The staining protocol was consistent with the description of Guo et al. [37]. Briefly, 100 μL of the cell suspension was placed into a 5 mL tube. The cell suspension was stained with 5 μL of Annexin V-FITC (Cat: 51-65874X, BD, USA) followed by 5 μL of PI (Cat: 51-66211E, BD, USA) at 25°C in darkness. After 15-min incubation, 400 μl of 1× binding buffer were added, and the stained cells were evaluated by a flow cytometry (BD FACS Calibur) within 40 min of preparation. Flow cytometry data analysis was performed using the ModFit LT v3.0 application software.

### Statistical analysis

All data were expressed as mean ± standard deviation. The statistical differences between the three experimental groups and control group were compared using one-way analysis of variance (SPSS 17.0 statistical software). P-value below 0.05 was considered statistically significant.

### Ethics statement

The animal protocols and all procedures of the experiment were performed in compliance with the laws and guidelines of Animal Care and Use Committee, Sichuan Agricultural University (Approval No: 2012- 024).
